# Using 3D Printing Technology to Teach Cartilage Framework Carving for Ear Reconstruction

**DOI:** 10.3389/fsurg.2020.00044

**Published:** 2020-07-17

**Authors:** Thomas H. Jovic, Emman J. Combellack, Zita M. Jessop, Iain S. Whitaker

**Affiliations:** ^1^Reconstructive Surgery and Regenerative Medicine Research Group, Swansea University, Swansea, United Kingdom; ^2^Welsh Centre for Burns and Plastic Surgery, Morriston Hospital, Swansea, United Kingdom

**Keywords:** 3D printing, andragogy, surgery, simulation, ear reconstruction

## Abstract

**Objective:** The aim of this study was to determine the validity of using a carvable 3D printed rib model in combination with a 3D printed auricular framework to facilitate the teaching, training and planning of auricular reconstruction.

**Design:** 3D printed costal cartilages from ribs 6–9 were produced using a FormLabs Form3 Printer and used to make negative molds. 2:1 silicone-cornstarch mixture was added to each mold to make 12 simulated 6–9th costal cartilages suitable for carving. 3D printed auricular frameworks were produced in polylactic acid using an Ultimaker 3 3D printer to demonstrate the component parts and constructed framework of an auricular reconstruction.

**Participants:** Twelve plastic surgery trainees attended a workshop in which they each attempted auricular reconstruction using the carvable models and 3D printed plastic models as a guide. All candidates completed a pre- and post-training questionnaire to assess confidence and comprehension of auricular reconstruction, and the suitability of the models for facilitating this teaching.

**Results:** Only 42% of trainees (*n* = 5) had observed an ear reconstruction in theater prior to the training course. Statistically significant improvements in the appreciation of the different components that make an auricular framework (*p* < 0.0001) and confidence in carving and handling costal cartilage (*p* < 0.0001) were noted following completion of the training. Highly significant improvements in comprehension of the approach to ear reconstruction (*p* = 0.006) and locating the subunits of a reconstructed ear from costal cartilage (*p* = 0.003) were also noted. 100% of participants felt the 3D printed teaching aids directly enhanced their learning.

**Conclusions:** Ear reconstruction is a complex, time consuming multi-stage operation demanding significant amounts of experience, planning and an appreciation of the 3D chondrocutaneous structure. In this study we have demonstrated the value of 3D printing in producing a suitable simulated costal cartilage model and as an adjunct to comprehending and planning a framework for auricular reconstruction.

## Introduction: Background and Rationale

Abnormalities of the auricle encompass a spectrum of partial to complete defects, acquired through both congenital, and acquired etiologies. At the extreme end of the spectrum of auricular anomalies are microtia and anotia: absence of part or entirety of the external ear, occurring in ~2 per 10,000 live births (1).

In expert hands, autologous ear reconstruction gives excellent results with relatively low complication rates (2). However, few surgeons acquire the necessary superspecialisation and caseload to effectively develop this expertise. The marked disparity in availability of this service, potentiated by the variable global incidence of auricular anomalies such as microtia (1) underpin the heterogeneity in surgical expertise. The degree of malformation is variable, reflecting disparities in the size, position, orientation and shape of the pinna which in turn demands highly personalized surgical intervention (3). The complexity and variability of autologous auricular reconstruction means outcomes rely heavily on the experience of the operating surgeon. As such, there has been significant interest in developing models to facilitate the planning, teaching, and training of autologous auricular reconstruction (4). The use of simulation as a training tool is crucial to improving skills, increasing precision while ensuring effectiveness and safety of patient care (5). This is particularly important in auricular reconstruction, where a physical 3D model can provide an improved understanding of the 3D architecture of the ear pre-operatively in order to re-create it through free hand carving (6).

An array of carvable materials have been sought in an attempt to emulate the uniquely carvable, yet elastic properties of costal cartilage. In 2016, Berens et al. (7) devised a low-cost, flexible and carvable composite material comprised of cornstarch and silicone sealant that was felt to closely resemble the desired properties of costal cartilage, such as firmness, carvability and bend required for surgical simulation. Previously explored options have included plant matter such as apples, potatoes and rubber (8, 9); animal (10, 11) and human cadaveric cartilage tissue (4) and synthetic materials such as polyurethane and silicone (7, 12, 13). There are limitations that hinder the use of many of these materials, including cost, inadequate replication of the mechanical properties and ethical considerations.

The search for the optimal material is only part of the challenge of auricular cartilage simulation. True emulation of the cartilage acquired intraoperatively requires the cartilage model to be presented to the surgeon in the shape, depth and orientation anticipated from a costochondral rib harvest. The value of 3D printed models in surgical education is well-characterised (14), and enables visual, tactile and spatial interaction with the learning content which may be of particular value to the visual and kinaesthetic learning styles that dominate in medical cohorts (15). The use of 3D printing technology has been revolutionary in enabling customized constructs to be developed based on patient-acquired radiographic imaging (CT, MRI) which can be readily converted into 3D image files for 3D printing purposes (16, 17). Not only does this hold high value for patient-specific pre-operative surgical planning, but the availability of this anatomical information allows for realistic and accurate simulation materials to be generated. Combining 3D printing technology with realistic simulated cartilage materials has the potential to significantly impact how surgeons develop the carving and planning skills that underpin autologous auricular reconstruction.

## Pedagogical Frameworks and Principles

The study was designed with the UK Higher Education Academy Professional Standards Framework born in mind, in particular, drawing on the principles within “Core knowledge,” “Professional Values,” and “Areas of Activity” to maximize the educational value of novel learning technologies to teach and support learning (18). To truly appreciate the value of novel 3D printed constructs in teaching ear reconstruction requires an appreciation of andragogy and learning styles in surgical trainees. The VAK model describes the three main learning types: Visual, Auditory, and Kinaesthetic (19) and coupling a student's learning style with a particular teaching method is believed to enhance learning and performance. As such, the production of simulated, carvable costochondral models enable visual, tactile and spatial interaction with the learning content which may be of particular value to visual and kinaesthetic learning styles. A study of 230 medical students identified that 45% were visual learners, 36% were auditory learners and 19% were kinaesthetic (15). The predominance (64%) of visual-kinaesthetic learners in medical cohorts indicates value for three dimensional models and simulation, which may augment learning and understanding of complex visuospatial concepts such as planning and performing autologous ear reconstruction, especially when used to enhance conventional didactic teaching methods. Having the opportunity to design an ear in 2D using a transparent film encourages participants to plan the component pieces based on an actual human auricle, before transferring their template onto a simulated rib. This exercise also affords participants the opportunity to plan out the best use of the available cartilage to yield the required number of ear components.

## Learning Environment

### Production of 3D Printed Molds for Rib Cartilage Models

**Figure 1:**
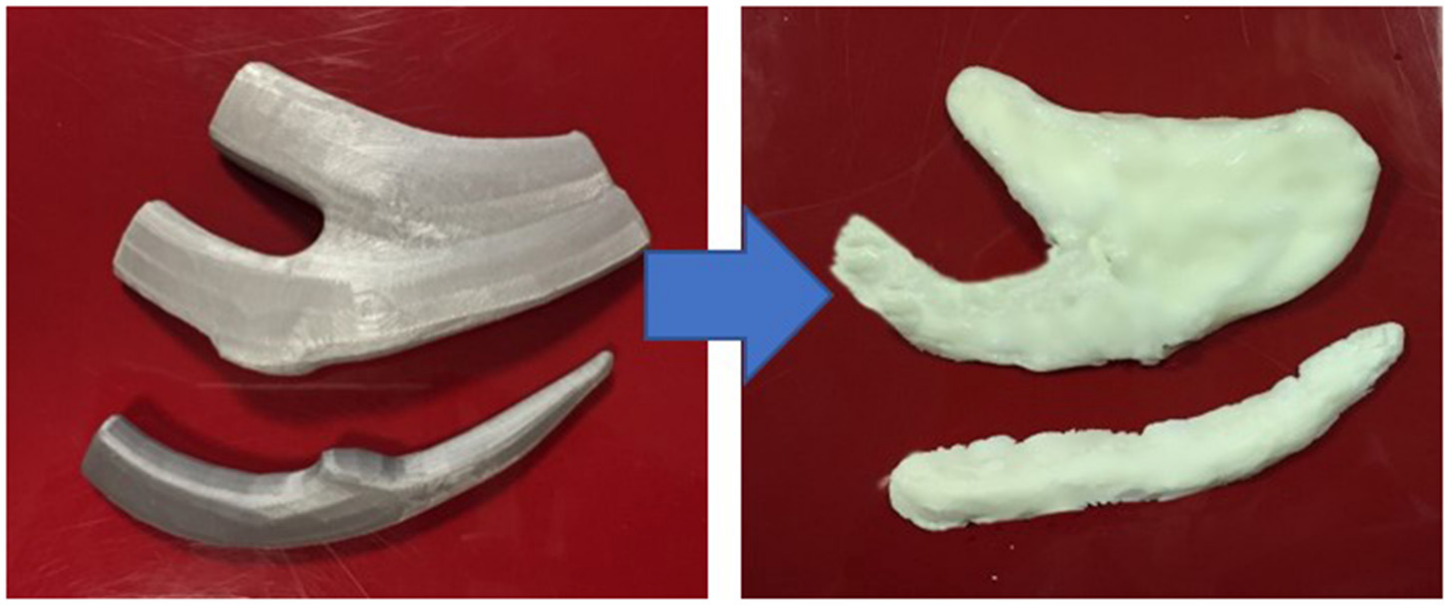
Left: 3D Printed costal cartilages 6–8 in Polylactic Acid [Ultimaker 3, Netherlands]; Right: Corresponding costal cartilages 6-8 in silicone:cornstarch 2:1 mixture.

Open-source Computed Tomography (CT) scan data was extracted to make 3D image files of costal cartilage from the sixth to ninth ribs using 3D image editing software (Autodesk Maya). The costal cartilages were exported as stereolithography files and printed in polylactic acid using an Ultimaker (3) Extrusion 3D Printer (Utrecht, Netherlands) or in resin using a FormLabs Form 2 Stereolithography 3D printer (MA, USA). The 3D printed costal cartilages were then used as impressions to make a rubber silicone mold for further rib constructs to be made (Figure 1).

### Production of Simulated Costal Cartilages 6–9

A search of the literature was undertaken to identify candidate materials for a carvable rib model using the PubMed, Embase and Google Scholar databases between 1980 and 2019. On the basis of practitioner validation and cost efficacy, a combination of cornstarch and industrial silicone sealant as originally described by (7) in a ratio of 1:2 cornstarch: silicone was used as the rib material. This mixture was added to the molds and allowed to set for 20 min, generating 12 separate costal cartilage carving models (Figure 1).

### 3D Printed Teaching Adjuncts

**Figure 2:**
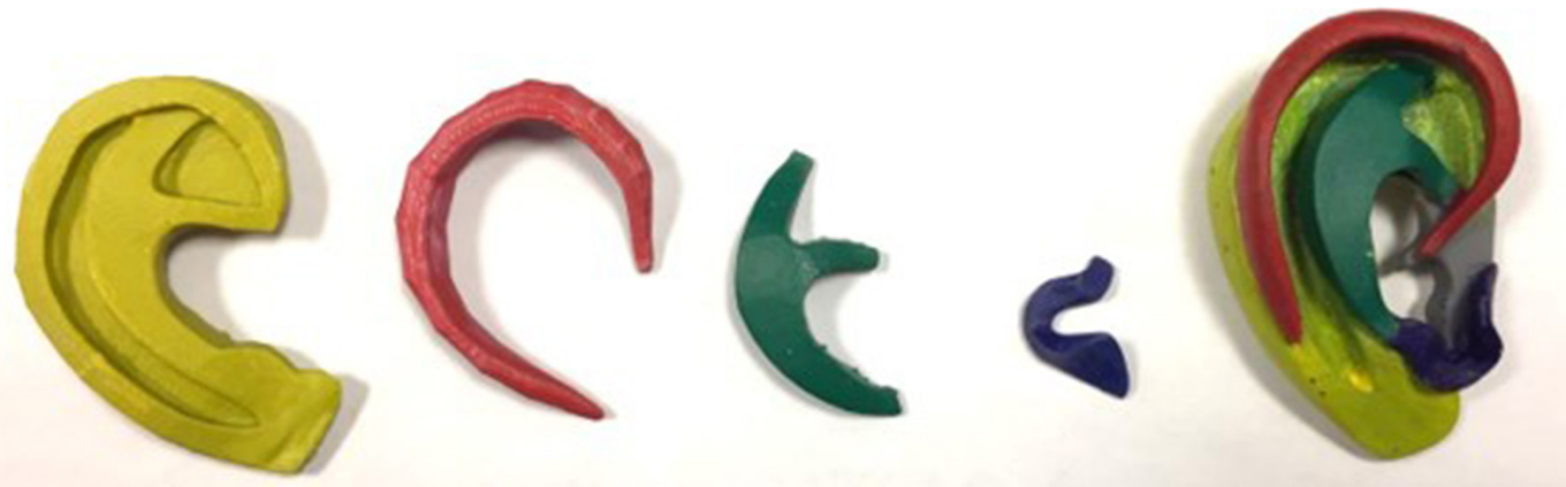
3D printed components of a “Firmin ear” for auricular reconstruction; combined in right of image.

Components of an autologous ear were generated using Autodesk Maya software according to the Firmin method of autologous ear reconstruction (20) including a base plate, antihelix, helix and tragus-antitragus complex. Dimensions and measurements were acquired through extraction from the previously created costal cartilage 3D image files. These constructs were colored individually with acrylic paint alongside a correspondingly colored, assembled Firmin ear to indicate their 3D spatial arrangement and interplay as combined components of a Firmin ear (Figure 2).

### Trainee Participation and Feedback

Twelve trainee plastic surgeons from Senior House Officer level (CT1) to senior registrar (ST8) attended a 3-h training workshop in which a video outlining the approach to auricular tracing, component extraction and carving was detailed as per the Firmin method of auricular reconstruction (20). Each trainee was given the simulated costal cartilage constructs, along with a colored, 3D printed Firmin ear and its separate components, three squares of transparent film and a 15-blade scalpel. Superglue was provided to affix the separate components in an assembled 3D auricular construct. Likert-scale questionnaires were provided to trainees to ascertain their prior knowledge and confidence in planning and performing auricular reconstruction prior to the course and the same questions provided immediately after delivery of the course. An additional Likert scale was provided to assess the candidates' experience with the simulated costal cartilage material in terms of texture, firmness, carving, bending and shape. The Likert scale values given by each participant were recorded and the mean and standard deviation calculated. Two-tailed paired student *t*-tests were performed to look for statistically significant changes (*p* < 0.05) in their comprehension, planning and performance of an autologous auricular reconstruction prior to and after completing the course.

## Objectives

The objectives of this study were to:

a) Validate the use of the silicone:cornstarch material described by (7) for simulated cartilage carving.
b) Augment ear reconstruction teaching with 3D printed learning adjuncts.
c) Produce reproducible and realistic simulation models for total auricular reconstruction.
d) Determine the impact of 3D printed simulation materials on learning and comprehension in surgery.

## Results

Each of the 12 anonymized questionnaires were evaluated by comparing pre and post course scores to determine the efficacy of the simulated costal cartilage in facilitating understanding and development of the complex technique of auricular reconstruction. Of the 12 trainees that undertook the training session 100% (*n* = 12) fully completed the questionnaire. Preceding this course only 42% of trainees (5 out of 12) had observed an ear reconstruction in theater, and of those that had seen an ear reconstruction prior to the course 60% (3 out of 5) had seen two or more reconstructions. Despite this however there was not a significant divergence in the scores when comparing the individual candidate responses.

**Figure 3:**
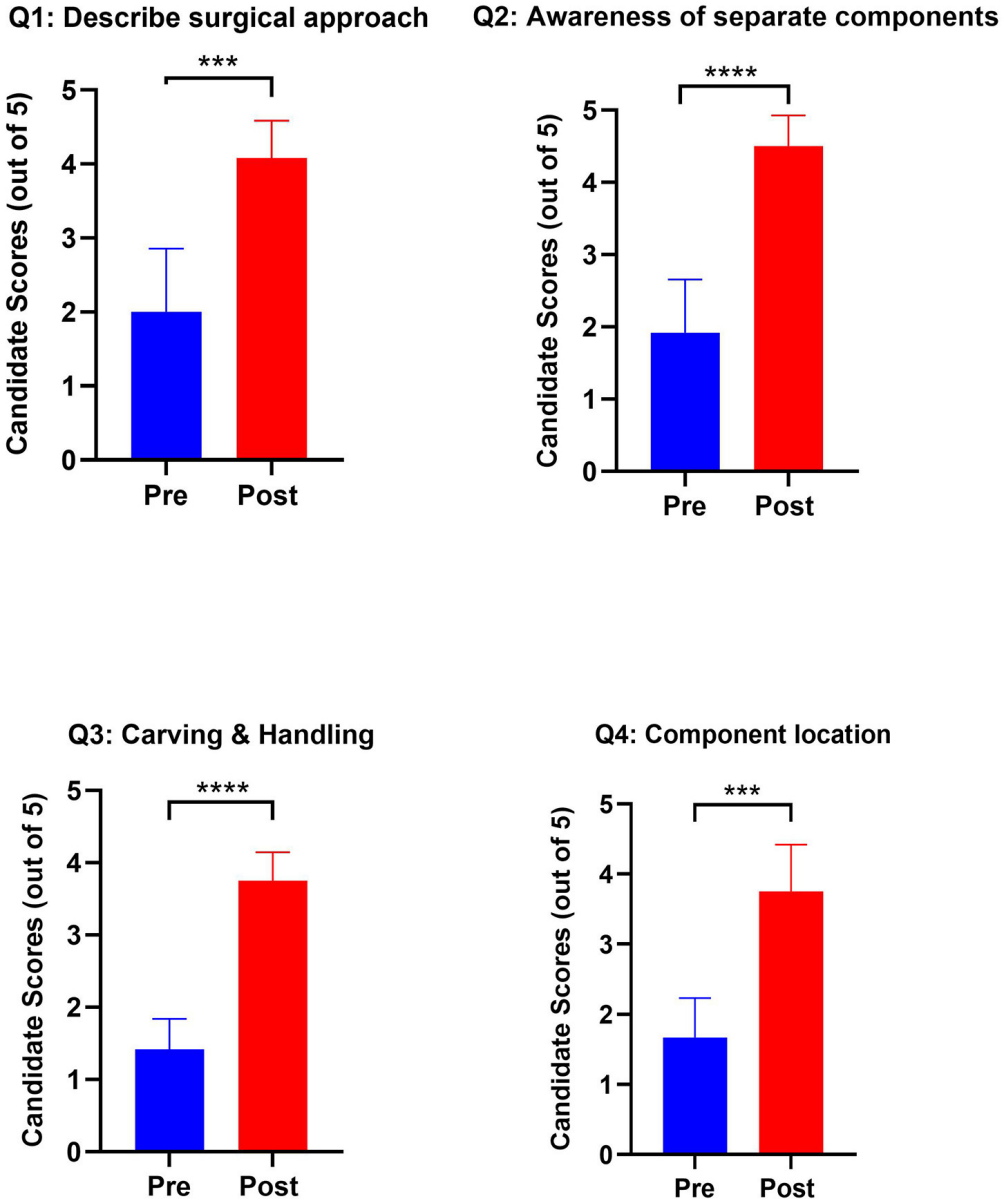
Graphs outlining pre- and post-training scores from candidates in their confidence and awareness of autologous ear reconstruction. *Paired Question 1*: I would feel able to describe the surgical approach to autologous ear reconstruction to an examiner or colleague. *Paired Question 2*: I am aware of the separate components needed to build an autologous ear from costal cartilage. *Paired Question 3*: I feel confident in carving and handling costal cartilage. *Paired Question 4*: I am aware of which components of the auricle I would harvest from each costal cartilage. (***p < 0.001; ****p < 0.0001).

**Figure 4:**
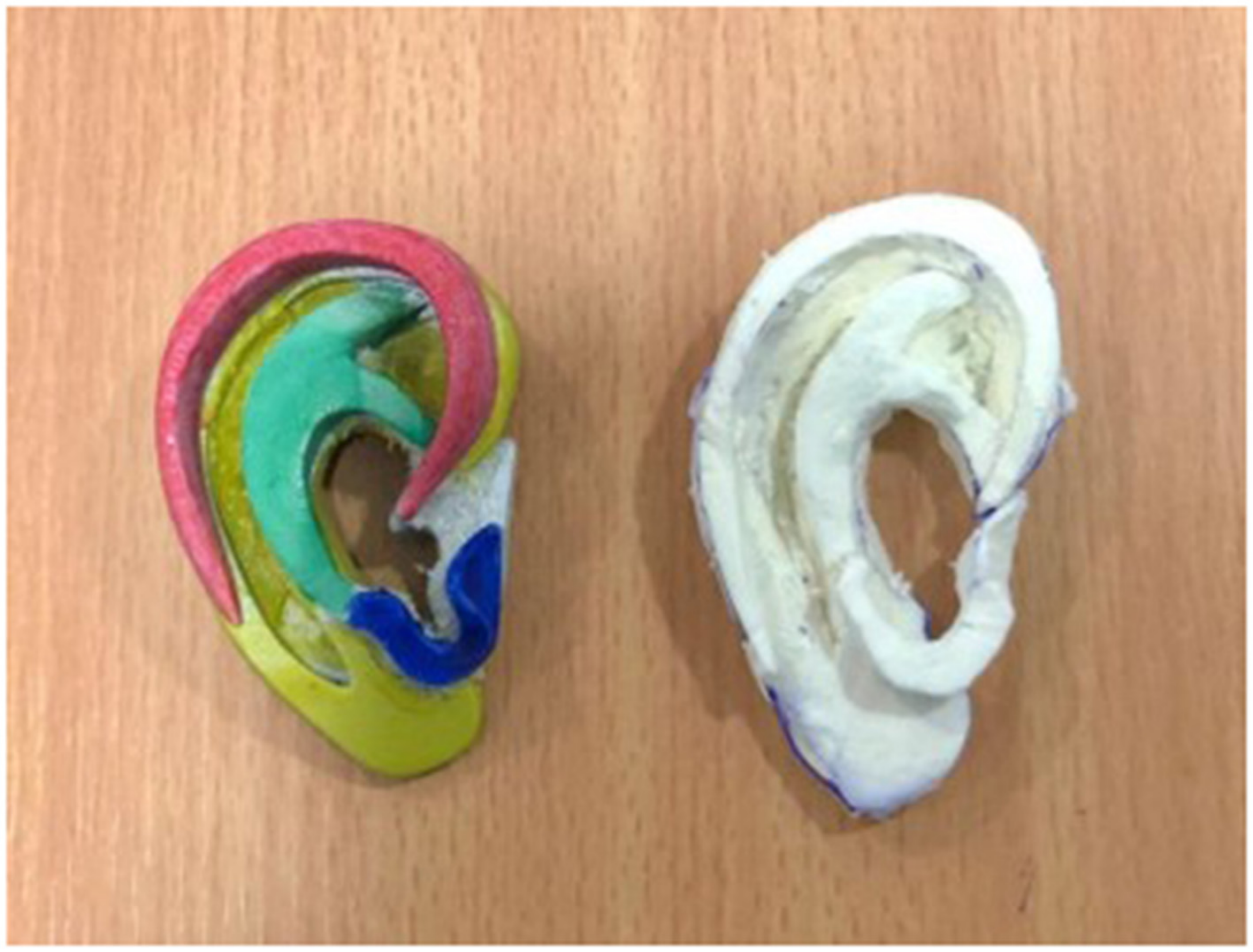
3D printed Firmin ear (left) and silicone:starch composite ear carved by participant (right).

Each of the four components assessed in the paired questionnaire (awareness of general approach, components of ear reconstruction, confidence in carving cartilage, confidence in producing separate components from each piece of costal cartilage) showed statistically significant improvement after completion of the course (Figure 3). Particularly stark was the appreciation of the different components that make a Firmin ear (Q2, *p* < 0.0001) and confidence in carving and handling costal cartilage (Q3, *p* < 0.0001). Highly significant improvements in comprehension of the approach to ear reconstruction (Q1, *p* = 0.006) and locating the subunits of a reconstructed ear from costal cartilage (Q4, *p* = 0.003) were also noted. Participants were able to use the visual aids to effectively produce accurate ear frameworks (Figure 4).

**Figure 5:**
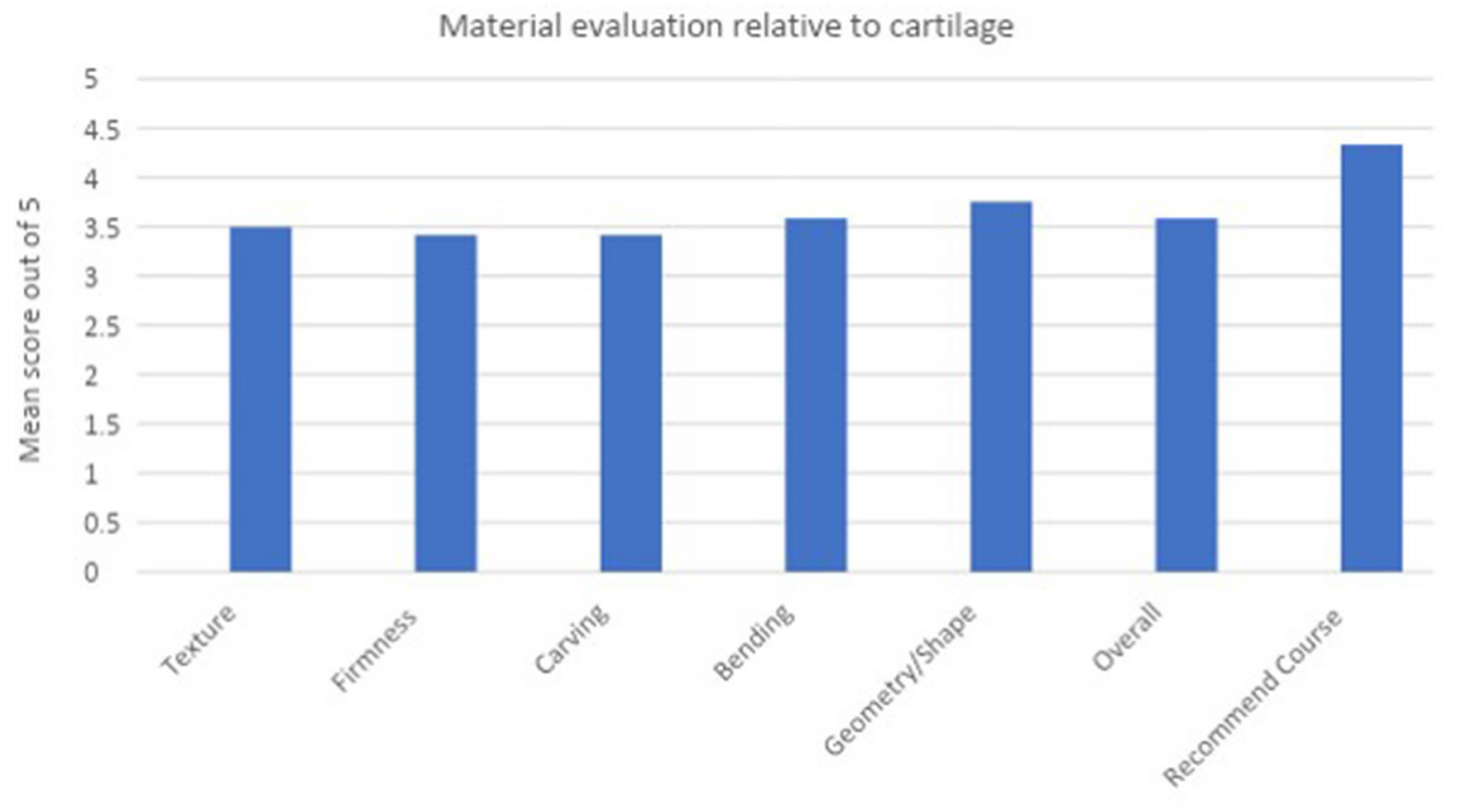
Bar chart to demonstrate participant ratings of the simulation costal cartilage material relative to cartilage.

The additional questions in the post course feedback focused on participant perception of course structure and how well it met the aims and objectives initially set, along with how relevant they found the content for future exams. All participants were asked to rate if they felt the 3D printed ear models [complete auricle, Firmin ear (combined) and Firmin ear (components)] improved their understanding of ear reconstruction and ultimately augmented their learning. 100% (12/12) participants agreed or strongly agreed with this statement with 11/12 participants choosing “5” on the Likert scale, indicating they strongly agreed that these models directly facilitated their learning (Figure 5).

Prior to the teaching session it was ascertained that 100% of the candidates had some experience handling cartilage in theaters through their previous operative experience and could therefore comment on this material as a direct comparison.

## Discussion

Auricular reconstruction is a time consuming, technically demanding and precise procedure, with no two auricular reconstructions being the same. Variability in rib anatomy, ossification pattern, reconstructive needs means the approach to auricular reconstruction is highly personalized and adaptable to match the heterogeneity of the general population (21, 22). The operative time can be highly variable depending on the quality and anatomy of the available cartilage, the mastery of which is a process of refinement with increased surgical experience. It has already been demonstrated that thorough pre-operative planning can reduce operative time, increase efficiency and evade complications in complex reconstructive surgery such as breast and craniofacial reconstruction (23, 24). The need for a reliable, reproducible and customisable simulation model to facilitate ear reconstruction has sparked interest in the pursuit of a realistic carvable material for surgical planning and training (3).

The interest initially appeared to favor material carvability above other mechanical properties, with readily accessible materials such as wood, apple, wax, and soap dominating early efforts (4, 8, 25). Though carvable, these materials are notoriously brittle and inelastic, failing to replicate the carvable nature of cartilage, and moreover lacking the necessary flexibility. This means that carving a helix, which would be conventionally bent into a curve is untenable. The shift in interest has since moved to finding a material that offers not only carvability but also a degree of flexibility and elasticity, with rubber, dental impression material and polyurethane being considered (6, 9, 13, 26). Whilst these materials offer a greater degree of flexibility, they suffer from key limitations. Rubber fails to replicate the geometry of costal cartilage and is unable to hold sutures, whilst dental silicone is expensive and difficult to carve (4, 7). One of the greatest potential strengths of synthetic materials is the ability to cast into specific shapes and sizes using 3D printed molds, offering the potential for specific models based on patient data.

The best emulators of native cartilage are cadaveric and animal costal cartilage. The major limitations of these approaches are the procurement, storage and preservation of tissue, as well as constraints surrounding ethics and availability (11). Technical limitations include calcification of elderly cadaveric cartilage, rendering the cartilage brittle and inflexible (27).

In 2016, Berens et al. described a compound containing cornstarch and silicone sealant, combined in a 1:2 ratio which was both carvable and flexible. The material was assessed by 2 experienced, blinded surgeons who rated the material 4 out of 5 overall on a Likert scale, and between 3 and 4 for properties such as texture, firmness, bending and carving (7). This material combination has since also been validated in producing simulated costal cartilage for airway reconstruction (28). The scores in our study were comparable to those observed in the original study of this material (with a higher *n* of 12).

By augmenting conventional teaching with the use of high-fidelity 3D printed ears, the participants were able to effectively visualize and recreate the individual components for autologous ear reconstruction using the Firmin technique (Figure 4). The use of 3D carved wax models as an adjunct to ear reconstruction teaching and surgical planning has been previously explored by Chen et al. (29), who has more recently used 3D printed ears derived from imaging data to produces templates for intraoperative planning (30, 31). 3D printing the ear separated into its component parts in this study, however, enabled participants to hold and compare their carved pieces directly to an ideal design, gaining a better appreciation how the separate components organize into a 3D auricle. That statistically significant improvements were observed in both understanding the principles and practice of auricular reconstruction supports the value of surgical simulation and the 3D printed reference materials used in this study.

## Conclusions

Autologous auricular reconstruction is one of the most complicated and conceptually challenging operations in Plastic and Reconstructive Surgery. The complexity of this reconstruction demands effective and realistic simulation models to hone cartilage carving skills, conceptualization and planning and refinement of framework composition. This has implications not only for trainees attempting to learn the procedure, but also established surgeons who which to exploit 3D printing technology for pre-operative planning. In this study we have demonstrated the value of 3D printing in producing simulated costal cartilage and as an adjunct to comprehending and planning a framework for auricular reconstruction.

## Limitations and Constraints

Our personal experience of this material is that where more uniform mixing was achieved, satisfactory carving and bending of the components such as the helix was achievable (Figure 4). In some of the models produced, heterogeneity in the mixing of the cornstarch and silicone did lead to some parts of the construct being more brittle than elsewhere.

The final component of ear reconstruction training is to observe how the construct appears under a pocket of skin (as *in vivo*). This was not a component of this study, yet the appearance of the constructs beneath a skin or fascial envelope has been explored previously by (20), resulting in the development of the Firmin Trainer (32). This contraption subjects an auricular framework, constructed from foam, to be placed under a rubber cap to which suction is applied, simulating the draping of skin over the ear (33). The incorporation of this methodology into further teaching sessions would augment the ability of learners to assess the aesthetic of their reconstructed ears, acquire visual feedback on their technique and verify the durability of their ear carvings.

## Data Availability Statement

All datasets generated for this study are included in the article/supplementary material.

## Ethics Statement

Ethical approval was not sought for this pedagogical study outlining a novel teaching methodology using 3D printed models. No patients or animal subjects were included to whom an intervention could be applied. All participants attended the teaching course voluntarily and feedback was acquired anonymously with consent from all participants.

## Author Contributions

TJ and IW conceived and designed the experiments and teaching session and prepared the aforementioned learning materials. TJ, EC, ZJ, and IW ran the teaching session and collected the data from questionnaires. TJ and EC were responsible for analyzing the data. TJ, EC, and ZJ drafted the manuscript which was edited and revised by IW. All authors contributed to the article and approved the submitted version.

## Conflict of Interest

The authors declare that the research was conducted in the absence of any commercial or financial relationships that could be construed as a potential conflict of interest.
